# Casting Light on the Adaptation Mechanisms and Evolutionary History of the Widespread Sumerlaeota

**DOI:** 10.1128/mBio.00350-21

**Published:** 2021-03-30

**Authors:** Yun Fang, Yang Yuan, Jun Liu, Geng Wu, Jian Yang, Zhengshuang Hua, Jibin Han, Xiying Zhang, Wenjun Li, Hongchen Jiang

**Affiliations:** a State Key Laboratory of Biogeology and Environmental Geology, China University of Geosciences, Wuhan, China; b State Key Laboratory of Desert and Oasis Ecology, Xinjiang Institute of Ecology and Geography, Chinese Academy of Sciences, Urumqi, China; c State Key Laboratory of Biocontrol, Guangdong Provincial Key Laboratory of Plant Resources and Southern Marine Science and Engineering Guangdong Laboratory (Zhuhai), School of Life Sciences, Sun Yat-Sen University, Guangzhou, China; d Department of Biological Sciences, Dartmouth College, Hanover, New Hampshire, USA; e Key Laboratory of Salt Lake Geology and Environment of Qinghai Province, Qinghai Institute of Salt Lakes, Northwest Institute of Eco-Environment and Resources, Chinese Academy of Sciences, Xining, China; CEH-Oxford

**Keywords:** ancestral state reconstruction, adaptation mechanisms, harsh environments, refractory organic compounds, Sumerlaeota

## Abstract

Sumerlaeota is a mysterious, putative phylum-level lineage distributed globally but rarely reported. As such, their physiology, ecology, and evolutionary history remain unknown. The 16S rRNA gene survey reveals that Sumerlaeota is frequently detected in diverse environments globally, especially cold arid desert soils and deep-sea basin surface sediments, where it is one dominant microbial group. Here, we retrieved four Sumerlaeota metagenome-assembled genomes (MAGs) from two hot springs and one saline lake. Including another 12 publicly available MAGs, they represent six of the nine putative Sumerlaeota subgroups/orders, as indicated by 16S rRNA gene-based phylogeny. These elusive organisms likely obtain carbon mainly through utilization of refractory organics (e.g., chitin and cellulose) and proteinaceous compounds, suggesting that Sumerlaeota act as scavengers in nature. The presence of key bidirectional enzymes involved in acetate and hydrogen metabolisms in these MAGs suggests that they are acetogenic bacteria capable of both the production and consumption of hydrogen. The capabilities of dissimilatory nitrate and sulfate reduction, nitrogen fixation, phosphate solubilization, and organic phosphorus mineralization may confer these heterotrophs great advantages to thrive under diverse harsh conditions. Ancestral state reconstruction indicated that Sumerlaeota originated from chemotrophic and facultatively anaerobic ancestors, and their smaller and variably sized genomes evolved along dynamic pathways from a sizeable common ancestor (2,342 genes), leading to their physiological divergence. Notably, large gene gain and larger loss events occurred at the branch to the last common ancestor of the order subgroup 1, likely due to niche expansion and population size effects.

## Abstract (precis)

In recent years, the tree of life has expanded substantially. Despite this, many abundant yet uncultivated microbial groups remain to be explored.

## INTRODUCTION

Microorganisms play a critical role in biogeochemical cycles and drive nutrient recycling and energy flow in nature (1). It is estimated that 85 to 99% of *Bacteria* and *Archaea* cannot yet be cultivated in the laboratory, drastically limiting researchers’ understanding of microbial life (2). Advances in culture-independent molecular techniques, especially metagenomics and single-cell genomics, have significantly expanded our knowledge of taxonomic, genetic, and metabolic diversity in various samples, such as soils, hydrothermal vents, and human bodies (3, 4). Such studies also allow us to better understand the ecological roles and interactions of ubiquitous uncultivated microorganisms and the origin and evolution of life (5–8).

The putative, monophyletic phylum-level lineage Sumerlaeota (formerly BRC1) is named after Sumerla, the underground goddess in Slavic mythology (9). Although genome fragments of Sumerlaeota were retrieved via genome-resolved metagenomics and single-cell genomics as early as in 2013 (3) and more draft genomes with high completeness are continuously reported (10), the physiology of Sumerlaeota was only inferred in 2019 on the basis of the first complete genome, “*Candidatus* Sumerlaea chitinivorans” BY40 (9). Metabolic reconstruction suggests a facultatively anaerobic, chemoorganotrophic lifestyle for BY40 based on the capability of utilizing carbohydrates (e.g., polysaccharides and chitin) and fermentation of organic substrates (9). These metabolic characteristics are consistent with the environmental conditions that BY40 inhabits, a deep subsurface thermal aquifer in the Tomsk Region of Western Siberia, Russia (9). Moreover, Sumerlaeota HGW-BRC1-1 from groundwater of the Hokkaido radioactive waste disposal site also contains a similar, complete chitinolytic pathway and has the potential for anaerobic hydrogenotrophic respiration (9, 10). However, it is believed that only the tip of the iceberg has been unveiled for this new bacterial lineage, especially considering that thousands of Sumerlaeota 16S rRNA gene sequences are deposited in the SILVA database while only two (nearly) complete genomes have been mined so far (9).

16S rRNA gene sequences assigned to Sumerlaeota were first discovered in anoxic bulk soil of flooded rice microcosms 19 years ago (11). Subsequently, Sumerlaeota were affirmed to be present in diverse natural and artificial environments, such as marine and freshwater sediments (12, 13), geothermal springs (14), deserts (15), activated sludge (16), and artificial wetlands (17). To the best of our knowledge, BRC1 bacterium clone P-8_B6, colonizing a cold arid desert soil of Mars Desert Research Station, USA, showed the maximum relative abundance (7.9%) to date (15), indicating that members of the Sumerlaeota are among the most abundant lineages in a microbial community and thrive in harsh environments.

Despite the abovementioned investigations, little is known about the physiology, ecology, and evolution of Sumerlaeota. To address these issues, we retrieved three nearly complete Sumerlaeota genomes from geothermal spring sediments in Tibet and another Sumerlaeota genome from saline lake sediment in Qinghai, China. The environmental distribution of Sumerlaeota taxa was depicted by analyzing 16S rRNA gene sequences from those new genomes and in the NCBI and IMG/M databases. Together with another 12 published genomes, the physiology and possible niches of these organisms were proposed. In addition, ancestral state reconstruction was performed to decipher the evolutionary history of Sumerlaeota.

## RESULTS AND DISCUSSION

### Genomic diversity and biogeography of Sumerlaeota.

**FIG 1:**
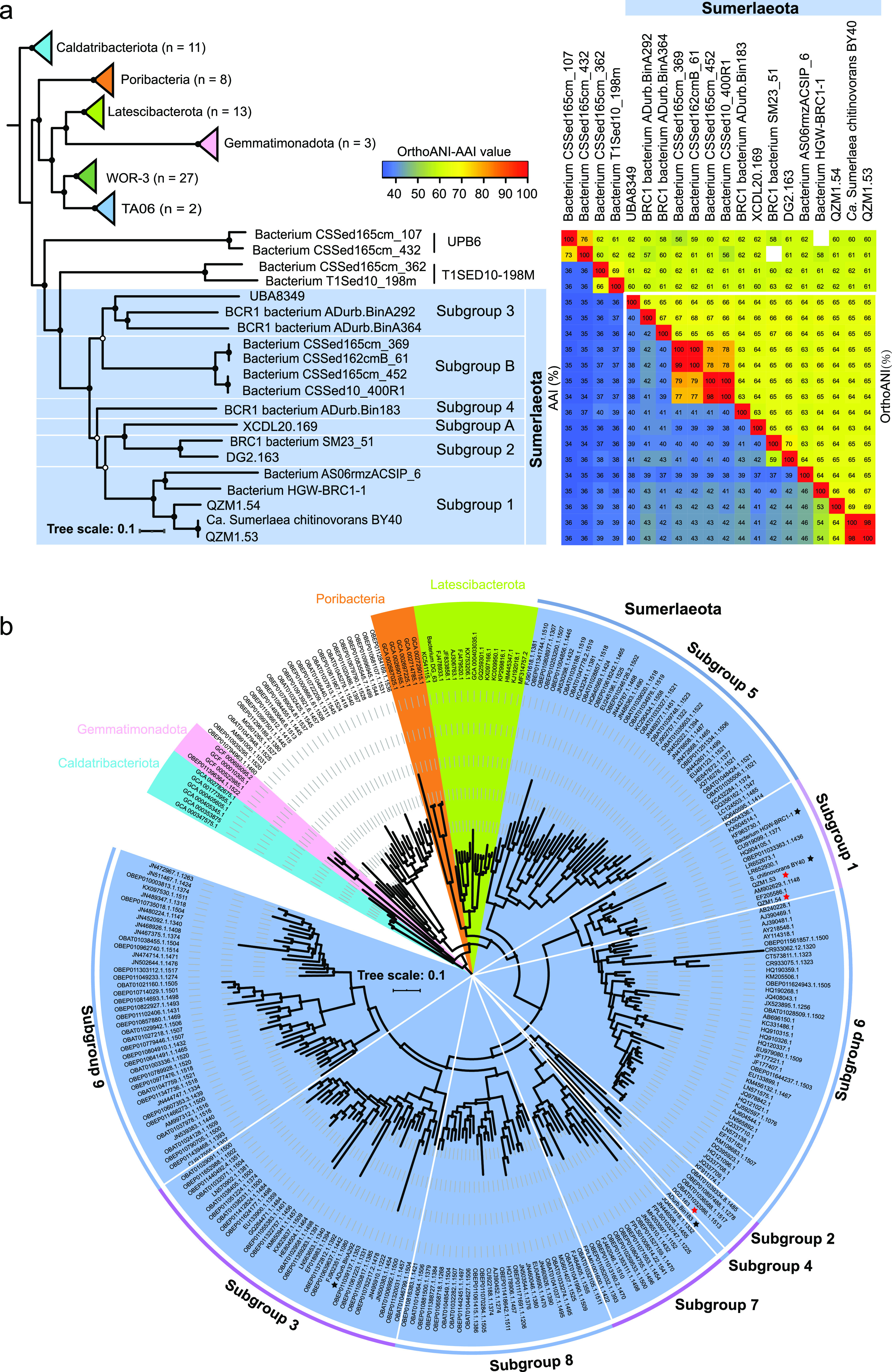
Phylogenetic placement of the Sumerlaeota MAGs. (a) Phylogenomic tree of Sumerlaeota, orthoANI, and AAI similarity values. This tree was constructed based on a concatenated alignment of 16 ribosomal proteins using IQ-TREE. These representative genomes were collected from the GTDB database. (b) Phylogenetic tree of the Sumerlaeota based on 16S rRNA gene sequences. The tree was also constructed using IQ-TREE. Ultrafast bootstrap values are based on 1,000 iterations, and percentages of ≥75% (50%) are shown using solid (hollow) circles. Different subgroups represent distinct orders according to the threshold of ∼83% 16S rRNA gene sequence similarity. Stars represent the available genomes.

To decipher the physiology and evolution of Sumerlaeota, the four MAGs retrieved in this study and another 12 MAGs published previously were analyzed (Table 1; see also Fig. S1 and S2 and Data Set S1a and b in the supplemental material). These genomes ranged from 1.88 to 3.77 Mb, with estimated completeness between 53% and 99% and less than 6% contamination. Considering the estimated average nucleotide identity (ANI) and average amino acid identity (AAI) values between these genomes and their relative evolutionary divergence (RED) values (Data Set S1c) (18, 19), phylogenomic analysis revealed that the 16 genomes were divided into six subgroups (orders) (Fig. 1a and Fig. S3), which was supported by the GTDB result (Data Set S1c). Interestingly, 16S rRNA gene-based phylogenetic analysis indicated that the Sumerlaeota was composed of nine subgroups at the ∼83% cutoff (Fig. 1b), suggesting that each subgroup represented one order according to the taxonomic classification criteria (20). Notably, due to the absence of 16S rRNA genes in some reconstructed MAGs, the resulting subgroups in the 16 riboprotein-based phylogeny could not completely match that in the 16S rRNA gene-based phylogeny. Thus, the five MAGs without 16S rRNA gene sequences were assigned to other groups, designated subgroup A (XCDL20.169) and subgroup B (bacterium CSSed165cm_369, CSSed162cmB_61, CSSed165cm_452, and CSSed10_400R1). In addition, topological differences were observed between the multiple marker gene-based and 16S rRNA gene-based phylogenies, which are obvious and common when comparing the difference between multiple-marker-gene-based and one-gene-based phylogenetic trees (21, 22). Currently, multiple marker gene-based phylogenomic trees are increasingly and widely used (8, 19).

**TABLE 1:** General characteristics of the Sumerlaeota genomes^*a*^

Genome ID	Completeness (%)	Contamination (%)	Total length (bp)	No. of scaffolds	No. of protein-coding genes	GC content (%)	Sample type (depth)	Habitat (pH, ^o^C, salinity)	Location
QZM1.53	99.37	6.18	3,252,853	14	2,580	56.04	Sediment (0–5 cm)	Geothermal spring (6.61, 64°C, NA)	Quzhuomu, Tibet, China
QZM1.54	98.81	6.18	3,109,332	17	2,508	54.44			
DG2.163	98.35	0	4,673,597	343	3,779	60.09	Sediment (0–5 cm)	Geothermal spring (8.79, 32°C, NA)	Daggyai, Tibet, China
XCDL20.169	75.06	3.23	3,646,393	487	2,960	57.48	Sediment (0–5 cm)	Saline Lake (8.09, 3°C, 70.7 g/liter)	Xiaochaidan Lake, Qinghai, China
*“Ca.* Sumerlaea chitinovorans” BY40	99.37	6.18	3,289,105	1	2,591	56.01	Groundwater (2,000 m)	Deep subsurface thermal aquifer (NA, 45°C, NA)	Tomsk region, Western Siberia, Russia
Bacterium HGW-BRC1-1	99.44	3.37	3,768,617	34	3,010	58.42	Groundwater (160 m)	Deep subsurface aquifer (7, 14–18°C, NA)	Horonobe Underground Research Laboratory, Hokkaido, Japan
BRC1 bacterium SM23_51	52.49	4.4	2,764,529	306	2,499	59.8	Sediment (24–32 cm)	White Oak River estuary sediment (NA, NA, NA)	White Oak River estuary sediment, NC, USA
Bacterium CSSed165cm_452	94.94	2.81	3,458,979	303	2,911	64.84	Sediment (0–5 cm)	Cock Soda Lake sediment (10.1, NA, 70 g/liter)	Kulunda Steppe, southwestern Siberia, Altai, Russia
Bacterium CSSed165cm_369	93.41	2.2	3,733,477	344	3,171	64.82			
Bacterium CSSed10_400R1	68.72	2.58	2,588,486	527	2,407	64.55	Sediment (0–5 cm)	Cock Soda Lake sediment (10.1, NA, 70 g/liter)	Kulunda Steppe, southwestern Siberia, Altai, Russia
Bacterium CSSed162cmB_61	82.25	2.35	3,495,477	454	3,070	64.9	Sediment (0–2 cm)		
UBA8349	67.11	0.07	2,991,443	486	2,886	53.35	Sediment (NA)	Wetland surface sediment (NA, NA, NA)	Twitchell Island, Sacramento Delta, CA, USA
ADurb.BinA292	85.08	2.2	3,543,516	505	3,107	66.62	Sludge (NA)	Anaerobic digester (NA, NA, NA)	Champaign-Urbana Sanitary District, IL, USA
ADurb.BinA364	64.41	2.75	4,114,461	1,718	4,342	63.22			
ADurb.Bin183	97.5	4.49	3,406,210	39	2,683	48.83			
AS06rmzACSIP_6	71.16	0	1,882,275	17	1,479	50.74	Sludge (NA)	Anaerobic digester (NA, NA, NA)	Seattle, WA, USA

a NA, not available.

10.1128/mBio.00350-21.1 TEXT S1 References in Data Set S1d. Download TEXT S1, DOCX file, 0.02 MB. Copyright © 2021 Fang et al. 2021 Fang et al. https://creativecommons.org/licenses/by/4.0/ This content is distributed under the terms of the Creative Commons Attribution 4.0 International license.

10.1128/mBio.00350-21.1 FIG S1 Geographic location of samples collected from Quzhuomu Hot Spring, Daggyai Hot Spring, and Xiaochaidan Lake. Download FIG S1, TIF file, 1.4 MB. Copyright © 2021 Fang et al. 2021 Fang et al. https://creativecommons.org/licenses/by/4.0/ This content is distributed under the terms of the Creative Commons Attribution 4.0 International license.

10.1128/mBio.00350-21.2 FIG S2 Environmental distribution of the Sumerlaeota through the 16S rRNA gene-based investigation. The environmental information of the sampling sites is described in Data Set S1c. Download FIG S2, TIF file, 9.6 MB. Copyright © 2021 Fang et al. 2021 Fang et al. https://creativecommons.org/licenses/by/4.0/ This content is distributed under the terms of the Creative Commons Attribution 4.0 International license.

10.1128/mBio.00350-21.3 FIG S3 Sumerlaeota phylogeny based on a concatenated alignment of 35 marker proteins. The tree was inferred with the LG+I+G4 mode in IQ-TREE, and ultrafast bootstrap values are indicated as solid circles (≥75%) and hollow circles (≥50% and <70%) at nodes. These representative genomes were collected from the GTDB database. Download FIG S3, EPS file, 1.3 MB. Copyright © 2021 Fang et al. 2021 Fang et al. https://creativecommons.org/licenses/by/4.0/ This content is distributed under the terms of the Creative Commons Attribution 4.0 International license.

10.1128/mBio.00350-21.7 DATA SET S1 (a) Physicochemical parameters of the sediment samples. (b) Information of the metagenomic datasets and assembly results. (c) GTDB classification of Sumerlaeota genomes. (d) Summarization of the Sumerlaeota 16S rRNA gene sequences published in previous studies. (e) Number of genes assigned to central metabolic pathways of the Sumerlaeota. (f) Number of genes encoding glycoside hydrolases (GHs) in the Sumerlaeota MAGs. (g) List of predicted number of gene families gained, lost, expanded, and contracted for the ancestral nodes and extant genomes. (h) The inferred gene gain and loss events at key nodes. (i) Analysis of covariance results of F-tests for ancestral compared to extant branches. (j) Linear regression relationships between these calculated rates of gene acquisition, loss, and duplication versus amino acid substitution rate. Download Data Set S1, XLSX file, 4.2 MB. Copyright © 2021 Fang et al. 2021 Fang et al. https://creativecommons.org/licenses/by/4.0/ This content is distributed under the terms of the Creative Commons Attribution 4.0 International license.

To better understand the ecological importance of Sumerlaeota, we attempted to describe their environmental distribution by using 16S rRNA gene-based analyses. Results revealed that Sumerlaeota were detected in 10 types of biotopes globally, including saline/hypersaline lakes, freshwater lakes, geothermal springs, deep subsurface aquifers, estuary/wetland sediments, bioreactors/artificial systems, oceans, soils/fields, deserts, and caves/sinkholes (Table 1, Fig. S2, and Data Set S1d), indicating the strong capability of these little-known microorganisms to adapt to both normal and harsh environments. Moreover, the tolerance of Sumerlaeota to the key environmental temperature, pH, and salinity allows this elusive bacterial lineage to occur in different (rather than in one particular) extreme environments. For instance, the highest growth temperature for Sumerlaeota is 64°C in Tibetan hot spring sediment, while the highest growth pH is 10.1 in southwestern Siberian Cock Soda Lake sediment, with ∼80 g/liter being the highest known salinity in the Guerrero Negro hypersaline microbial mat. Moreover, the relative abundance of Sumerlaeota was up to 1% and increased with the depth of microbial mats from a hypersaline evaporation pond in Guerrero Negro (12), suggesting a facultatively anaerobic lifestyle for this relatively abundant lineage. To our knowledge, Sumerlaeota was also similarly abundant in the thermophilic mat from one Tibetan geothermal spring (pH 7.0, 61°C), with a relative abundance of ∼1% (14). Most samples (>90%) were from inland biotopes that occur in mid-latitude regions, while a few were from marine biotopes. In deep-sea basin surface sediments of the South China Sea, Sumerlaeota could account for up to 6% of the bacterial 16S rRNA gene clones (23). Thus, Sumerlaeota may be of great environmental importance, considering that the subseafloor marine biosphere is one of the largest reservoirs of microbial biomass on Earth (24). These findings illustrated that the Sumerlaeota are global generalists, to some extent, acting as one of the core microbial lineages in some harsh environments with low nutrient availability. Such environmental distribution is consistent with physiological features of the Sumerlaeota inferred by genome analysis (described below).

### Physiological potential.

**FIG 2:**
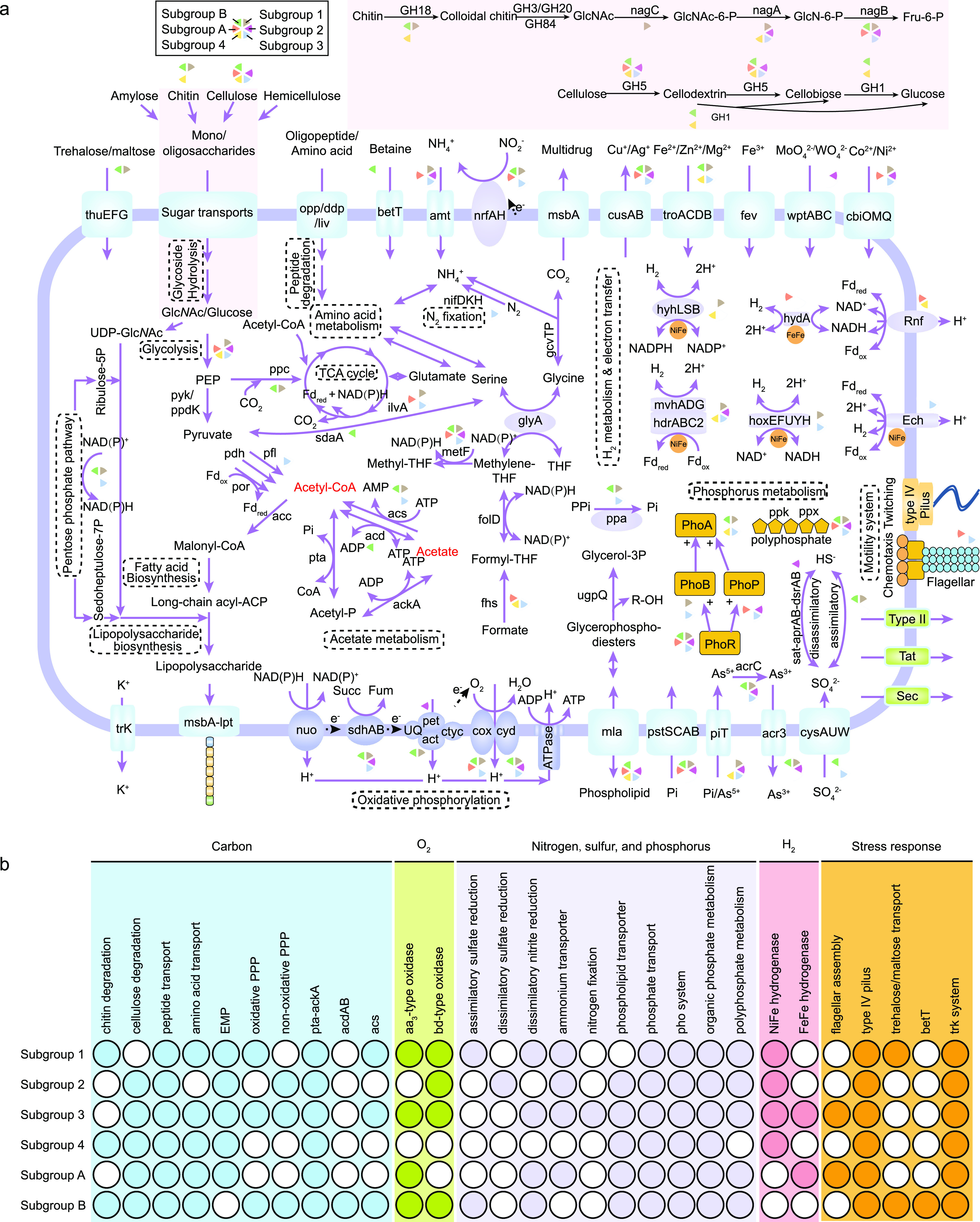
Metabolic pathways of the Sumerlaeota. (a) Metabolic reconstruction of the Sumerlaeota subgroups. (b) Metabolic characteristics of different Sumerlaeota subgroups. The copy number of genes in each genome is listed in Data Set S1e.

**(i) Core carbon metabolism.** The genome-scale metabolic reconstruction revealed that Sumerlaeota had the genetic potential to degrade detrital organic matters, including complex carbohydrates and proteins (Fig. 2 and Data Set S1e), implying a heterotrophic lifestyle for these organisms. Results showed that they could code for a series of enzymes capable of degrading amylose, chitin, cellulose, and hemicellulose (Data Set S1f), suggesting their roles in the initial degradation and hydrolysis of complex carbon compounds. Cellulose (a β-1,4-glucose polymer) and hemicellulose (polysaccharides consisted of xylose, arabinose, mannose, and galactose) were considered the two most abundant carbon sources in nature, which could be enzymatically hydrolyzed by only a few microorganisms (25, 26). Notably, genes encoding cellulose-active enzymes (e.g., beta-glucosidase, cellulase, and endoglucanase) affiliated with the GH1 and GH5 families and hemicellulose-active enzymes were detected in some Sumerlaeota members, implying their capacity of consuming cellulose and hemicellulose, which has not yet been reported for Sumerlaeota. Cellulose might first be hydrolyzed into short-chain cellulose/cellooligosaccharides and cellobiose by endoglucanase/cellulase of the GH5 family in members of subgroups 2, 3, 4, A, and B, and then these products would likely be transformed into glucose and glucose 6-phosphate, the substrates of Embden-Meyerhof-Parnas glycolysis, by the GH1 β-glucosidase in members of subgroups 4 and B (Fig. 2 and Data Set S1e). It also was notable that DG2.163, UBA8349, and XCDL20.169 harbored 13, 35, and 25 copies, respectively, of genes encoding hemicellulolytic enzymes (Data Set S1f), indicating that these Sumerlaeota members act as hemicellulose scavengers in nature.

**FIG 3:**
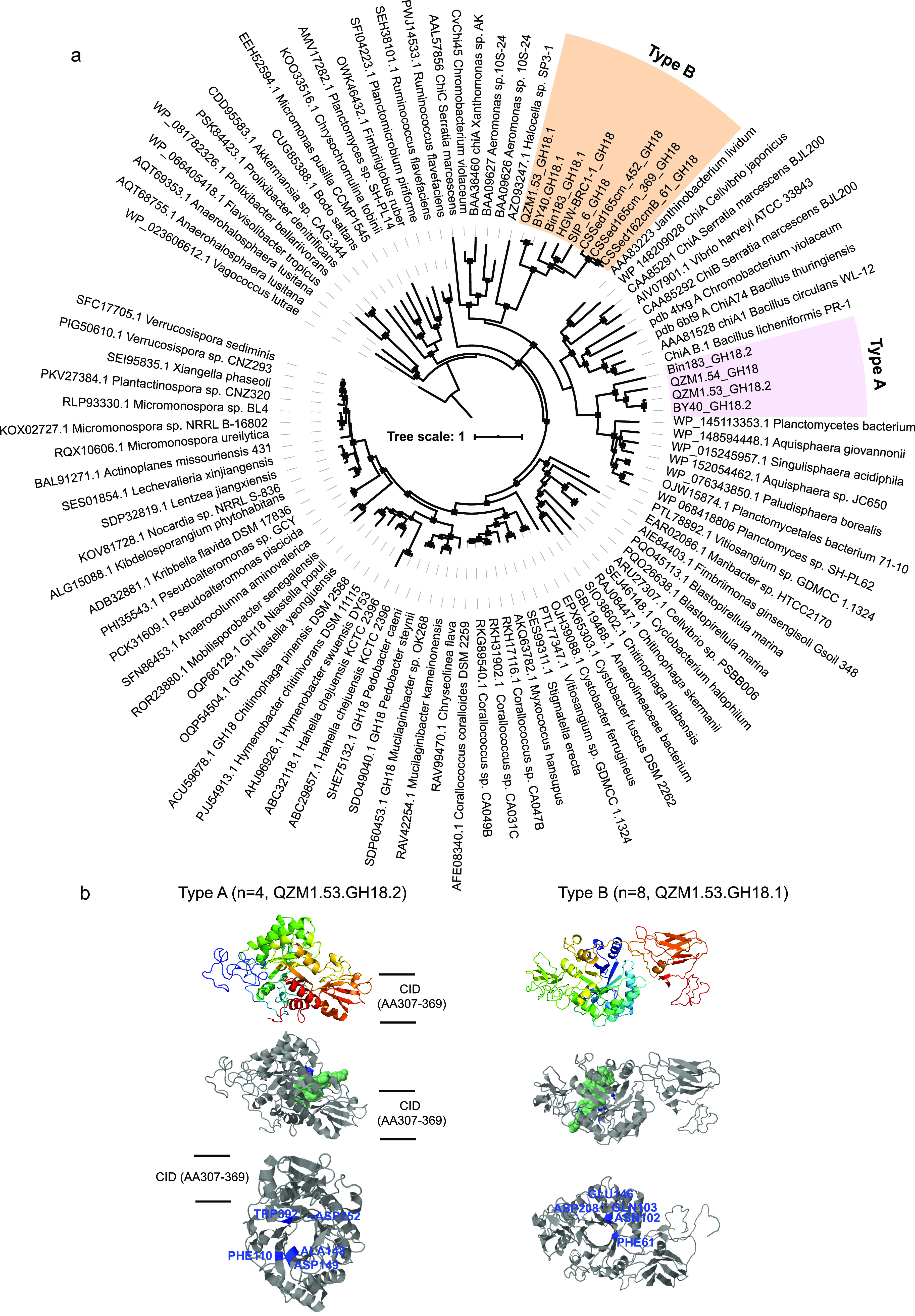
Phylogeny and predicted structure of Sumerlaeota chitinases. (a) Phylogenetic tree of the GH18 family chitinases. Sumerlaeota populations contain two types of chitinases. Ultrafast bootstrap values are based on 1,000 iterations, and percentages of ≥75% are shown using solid squares. (b) Predicted structure of type A and B chitinases from the Sumerlaeota. Orthogonal views of type A and B chitinase monomers, colored from the N terminus (blue) to the C terminus (red), respectively, are shown. The bound molecule is presented using a green space-filling model, revealing the position of the substrate binding groove. CID represents the insertion domain of chitinase.

Chitin is one of the most abundant biopolymers widely distributed in nature and interacts with both carbon and nitrogen cycles (27). Research revealed that members of subgroups 1, 4, and B are potential chitin degraders due to the presence of the complete chitinolytic pathway. Specifically, the initial hydrolysis of the (1→4)-β-glycoside bond of chitin is likely performed by chitinase affiliated with the GH18 family, resulting in colloidal chitin that is further split into dimers. Subsequently, β-N-acetyl-hexosaminidase of the GH3, GH20, or GH84 families could cleave the generated dimers into monomers like *N-acetyl-d-*glucosamine (GlcNAc). The resulting GlcNAc then is phosphorylated by *N*-acetylglucosamine kinase into GlcNAc-6-P, which is further deacetylated by *N*-acetylglucosamine-6-phosphate deacetylase with the production of glucosamine-6-phosphate. Finally, the resulting glucosamine-6-phosphate is converted by glucosamine-6-phosphate deaminase into fructose-6-phosphate that enters Embden-Meyerhof-Parnas glycolysis (Fig. 2 and Data Set S1e). Phylogenetic analyses also showed that these organisms contained two types of chitinases, including type A (*n *=* *4) and B (*n *=* *8) (Fig. 3). Type A chitinase possessed one signal peptide, followed by the GH-18 catalytic domain composed of the triosephosphate isomerase (TIM) barrel (α/β)_8_ domain and the chitinase insertion domain (CID) (Fig. 3b). To our knowledge, the CID was found only in subfamily A of family 18 chitinases, sandwiched between the seventh and eighth β-strands of the TIM barrel fold of the catalytic site (28). These processive enzymes permit the substrate to be threaded through the tunnel, catalyzing it without being detached. The processivity of chitinases and some aromatic residues (such as Phe and Trp) in the substrate-binding groove is considered beneficial for the hydrolysis of crystalline chitin (23). Different from type A chitinase, type B chitinase only contain one signal peptide and the characteristic (α/β)_8_-TIM-barrel catalytic region (Fig. 3b). These nonprocessive enzymes without a CID domain might have more shallow and open clefts, providing more flexibility within the catalytic site that enables detachment and reattachment in disordered regions of the chitin polymer. Intriguingly, QZM1.53 and “*Candidatus* Sumerlaea chitinovorans” BY40 of subgroup 1 and ADurb.Bin183 from subgroup 4 had both types of chitinases, which enable the organisms to have a broader substrate spectra than those Sumerlaeota members with either type A or B chitinase. A similar finding had also been observed in Serratia marcescens (28, 29). Despite a pure culture of any Sumerlaeota species not having been obtained until now, *in vitro* expression of the chitinase gene from “*Candidatus* Sumerlaea chitinivorans” BY40 has been performed and chitinolytic activities and substrate specificity pattern of this purified protein are confirmed, consistent with the above-mentioned inference (9). Overall, the hydrolytic potential for organic substrates (such as cellulose and chitin) varied among different subgroups, implying their distinct niches.

All the Sumerlaeota MAGs contained genes encoding amylolytic enzymes, but the corresponding genes might be different. For example, α-amylases of the GH13 and GH57 families and the GH77 amylomaltase were widely employed, yet amylases affiliated with the GH15 and GH97 families were found in only three and four Sumerlaeota MAGs, respectively. Moreover, more than 60 kinds of peptidases were identified in Sumerlaeota MAGs, suggesting that proteinaceous compounds are alternative carbon sources and electron donors for this lineage (Data Set S1e). Considering that these studied Sumerlaeota colonized oligotrophic environments (with 0.27 to 1.09% total organic carbon [TOC]), the hydrolytic potential for organic substrates (especially refractory substrates) enabled them to be more advantageous than other microorganisms. Meanwhile, the degradation of refractory organic matter catalyzed by Sumerlaeota could provide bioavailable organic carbon to other heterotrophs, implying the importance of Sumerlaeota in maintaining community stability under harsh conditions. These degradation products might be further oxidized and assimilated via Embden-Meyerhof-Parnas glycolysis, the pentose phosphate pathway, and the tricarboxylic acid (TCA) cycle. In these processes, Sumerlaeota appeared to be able to decarboxylate pyruvate to acetyl-coenzyme A (CoA) to link glycolysis with the TCA cycle through pyruvate ferredoxin/flavodoxin oxidoreductase (*por*) under anoxic conditions but through pyruvate dehydrogenase (*pdh*) under oxic conditions (Fig. 2 and Data Set S1e). Anaerobic and aerobic conditions usually shift in oxygen-limited environments such as deep subsurface aquifers and surface sediments (9, 30, 31). Thus, this physiological feature could be beneficial for Sumerlaeota to adapt to oxygen fluctuations.

Aside from the findings described above, we also found that the Sumerlaeota members might be able to metabolize acetate (Fig. 2 and Data Set S1e). In this study, eight of the sixteen Sumerlaeota MAGs coded for AMP-forming acetyl-CoA synthetase (ACS) involved in acetate utilization, and most contained the classical Pta-Ack pathway for acetate production/assimilation. This suggests that Sumerlaeota are acetate producers or consumers, depending on the oxygen concentration and/or oxidation reduction potential. A previous study also illustrated that members of subgroup 1 were able to convert acetyl-CoA to acetate via this Pta-Ack pathway (9). Interestingly, another acetogenesis pathway using reversible ADP-forming acetyl-CoA synthetase (ACD) was also present in subgroup B (CSSed165cm_452 and CSSed165cm_369). ACD was considered exclusive to *Archaea* until recently it was found in a few bacteria (32, 33). Increasing evidence illustrated that acetogenesis played an important role in organic carbon cycling in diverse (microaerobic or anaerobic) extreme habitats, such as deep subsurface (34, 35), hot springs (36, 37), and soda lakes (38–40). Collectively, these results demonstrate that Sumerlaeota harbor the potential to grow as acetogens and, thus, contribute to carbon cycles in these extreme ecosystems.

**(ii) Hydrogen metabolism.** Multiple hydrogenase genes were identified in the Sumerlaeota MAGs, suggesting that Sumerlaeota harbor the potential for H_2_ metabolism (9, 10). For example, three types of group 3 [NiFe]-hydrogenases (group 3b, 3c, and 3d) were found in at least two Sumerlaeota subgroups (Fig. 2, Fig. S4, Data Set S1e). The group 3b Ni,Fe-hydrogenase is widely distributed in Sumerlaeota and directly couples the oxidation of NADPH to evolution of H_2_. Note that some group 3b [NiFe]-hydrogenases also retain the sulfhydrogenase activity to reduce elemental sulfur (S^0^) to hydrogen sulfide (H_2_S) (41). The group 3c [NiFe] methyl-viologen-reducing hydrogenase (*mvhADG*) and heterodisulfide reductase (*hdrABC2*) form a functional complex that can simultaneously reduce ferredoxin and CoB-CoM heterodisulfide during H_2_ oxidation and has even been detected in hydrogenotrophic methanogens and some bacteria (e.g., Deltaproteobacteria) (42). However, due to the absence of CoM biosynthesis and methanogenesis pathways, this complex may be involved in energy-conserving metabolisms (such as the oxidation of inorganic sulfur compounds and the reduction of sulfate and ferric iron) in Sumerlaeota populations, as previously reported (43, 44). The oxygen-tolerant group 3d [NiFe] hydrogenase complex (HoxEFUYH) in Sumerlaeota has been proposed to maintain redox balance by interconverting electrons between NADH and H_2_ according to previous research (41). Moreover, only XCDL20.169 had the potential to reversibly bifurcate electrons from H_2_ to ferredoxin and NAD due to the detection of the group A3 [FeFe] hydrogenases and membrane-bound Rnf complexes (*rnfABCDEG*) (45, 46). The presence of group 1a respiratory H_2_-uptake [NiFe]-hydrogenase in HGW-BRC1-1 suggests that this population is capable of anaerobic hydrogenotrophic respiration (9). In addition, membrane-bound [NiFe] hydrogenases (group 4e, *echABCDEF*), involved in H_2_ and ferredoxin cycling, were only detected in UBA8349, suggesting an extra pathway of energy conservation in this species compared with other Sumerlaeota members (47). Interestingly, most detected hydrogenases, such as group 3c, 3d, and 4e [NiFe] hydrogenases and group A3 [FeFe] hydrogenases, are bidirectional; thus, we cannot rule out the possibility that Sumerlaeota produces H_2_ via anaerobic carbohydrate fermentation. Overall, these hydrogenases in Sumerlaeota likely are involved in redox homeostasis and energy conservation and supply intracellular reducing equivalents needed for various redox reactions.

10.1128/mBio.00350-21.4 FIG S4 Gene operons of hydrogenases in the Sumerlaeota. Download FIG S4, EPS file, 0.9 MB. Copyright © 2021 Fang et al. 2021 Fang et al. https://creativecommons.org/licenses/by/4.0/ This content is distributed under the terms of the Creative Commons Attribution 4.0 International license.

**(iii) Oxidative phosphorylation.** Subgroups 1, 2, 3, and B were identified to possess complete oxidative phosphorylation systems composed of NADH-quinone oxidoreductase (complex I, *nuo*), succinate dehydrogenase (complex II, *shdAB*), the quinol-oxidizing *bc*_1_/alternative complex (complex III, *pet*/*act*), *aa*_3_-type/*bd*-type cytochrome *c* oxidase (complex IV, *cox*/*cyd*), and F-type ATPase (complex V) (Fig. 2 and Data Set S1e). It is known that *aa*_3_-type cytochrome *c* oxidase is a low-affinity terminal oxygen reductase working under oxic conditions, whereas *bd*-type is a high-affinity terminal oxygen reductase capable of functioning under oxygen-limiting condition (48). The presence of both cytochrome *c* oxidases possibly enabled these subgroups (1, 3, and B) to thrive in environments with oxygen fluctuations. This hypothesis is also supported by the presence of *por* and *pdh* genes in Sumerlaeota, as mentioned above.

**(iv) Nitrogen, phosphorus, and sulfur metabolism.** The six-electron reduction of nitrite to ammonia is a crucial step in the biogeochemical cycle of nitrogen (93). The key *nrfAH* genes encoding nitrite reductase were widely distributed in members of subgroups 1, 3, A, and B, with the presence of *narGHI* genes (encoding nitrate reductase) in subgroups 3 and B (Fig. 2 and Data Set S1e), implying some Sumerlaeota perform complete dissimilatory nitrate reduction under anaerobic conditions. Moreover, it is intriguing that UBA8349 contains both *nifDKH* and *anfDKGH* genes, encoding molybdenum-iron and iron-iron nitrogenase for nitrogen fixation, respectively, which appears to be the first report in Sumerlaeota and expands the role of Sumerlaeota in the nitrogen cycle. As we know, nitrogen fixation occurs under anoxic conditions, as oxygen can deactivate nitrogenases. Thus, the flagellar motor and chemotaxis system identified in UBA8349 could help cells to migrate toward conditions that are amenable to growth (49, 50). These findings illustrate that Sumerlaeota may be an important supplier of organic nitrogen in extreme environments. In addition, DG2.163 likely harbors the potential to reduce sulfate to sulfide through a dissimilatory pathway, due to the detection of all important genes (*sat*, *aprAB*, *dsrAB*, *dsrC*, *dsrMKJOP*, and *qmo*) in the pathway. The concatenated DsrAB protein tree also supports this conclusion and reveals that the *dsrAB* genes belong to the unknown environmental supercluster 1 (51), whose origin is still a mystery (Fig. S5). Considering that sulfate is usually abundant in the geothermal springs (52), this ability to reduce sulfate is of great advantage for Sumerlaeota to survive.

10.1128/mBio.00350-21.5 FIG S5 Concatenated DsrAB protein tree of Sumerlaeota. Ultrafast bootstrap values of ≥75% (50%) are shown using solid (hollow) circles. Download FIG S5, EPS file, 1.9 MB. Copyright © 2021 Fang et al. 2021 Fang et al. https://creativecommons.org/licenses/by/4.0/ This content is distributed under the terms of the Creative Commons Attribution 4.0 International license.

For inorganic phosphorus utilization, the key *phoA* gene, which encodes a well-characterized alkaline phosphatase that hydrolyzes phosphate esters for assimilation (53), prevails in Sumerlaeota, except subgroups 2 and A (Fig. 2 and Data Set S1e). Notably, members of subgroups 1, 3, and B likely code for soluble inorganic pyrophosphatase (*ppa*), which can hydrolyze inorganic pyrophosphate (PPi) to orthophosphate and release a considerable amount of energy to support growth (54). PPi is a common by-product of biosynthesis (such as DNA, peptidoglycan, and other biopolymers) and is also produced during the posttranslational modification of proteins (55). Genes encoding polyphosphate kinase (*ppk* or *ppk2*) and/or exopolyphosphatase (*ppx*) also were detected in most of the Sumerlaeota except subgroup 4, suggesting that these organisms hydrolyze polyphosphate in phosphorus-deficient environments (56). These findings hinted that these elusive bacteria usually survive under phosphorus starvation conditions, which is supported by the ubiquitous presence of diverse phosphate transporters (PstSCAB and TC.PIT) in Sumerlaeota. Aside from inorganic phosphorus, almost all Sumerlaeota can perform organic phosphorus mineralization using glycerophosphoryl diester phosphodiesterase (UgpQ) (57, 58). In brief, these results showed a significant survival advantage for the mysterious Sumerlaeota to live in nutrient-limited niches.

### Metabolic adaptation to stress.

To protect from damage caused by extreme environmental stresses (e.g., high temperature and salinity), Sumerlaeota populations have developed a series of adaptation mechanisms (Fig. 2 and Data Set S1e). To resist salinity stress, members of subgroup B from a hypersaline soda lake may employ two membrane-based strategies: (i) relying on the influx of ions (such as potassium) from the surrounding environment (e.g., a “salt-in” strategy) to maintain pH and K^+^ homeostasis by using potassium uptake protein of the Trk family and (ii) accumulating low-molecular-weight compatible organic solutes (such as betaine and trehalose) to balance the external osmotic pressure (e.g., the “salt-out” strategy) through choline/glycine/proline betaine transporters (*betT*) and trehalose/maltose transporters (*thuEFG*). The use of a mixture of both strategies has been observed in the halophilic archaeon Haladaptatus paucihalophilus (59). Arsenic detoxification is ubiquitous in the biosphere. Some important genes involved in arsenic metabolism are detected in the Sumerlaeota genomes, including those encoding arsenate reductase (*asr*C), arsenite transporter (*acr3*), and ArsR family transcriptional regulator (*asrR*), suggesting that Sumerlaeota are capable of arsenic detoxification. Given that the oxygen concentration fluctuates drastically in environments where Sumerlaeota reside (9, 10, 60), a series of response proteins may be used to resist oxidative stress, such as superoxide dismutase (*sod*), superoxide reductase (*dfx*), thioredoxin reductase (*trxR*), and peroxiredoxins (*prxQ*) (61). Furthermore, these organisms harbored the *ppk*/*ppk2* and/or *ppx* genes, involved in polyphosphate biosynthesis and degradation, as mentioned above. Numerous studies have proven that polyphosphate plays a fundamental role in stress resistance for prokaryotes, such as (i) gaining energy from its degradation by polyphosphate kinase (*ppk*/*ppk2*), (ii) regulating the homoeostasis of heavy metals and other cations, and (iii) affecting gene expression and specific enzymatic activity and even promoting mutagenesis under stressful conditions, since it can mimic DNA to bind to RNA (61). Thus, polyphosphate not only modulates adaptive mechanisms that protect cells from diverse stresses (56) but also may participate in the adaptive evolution of microorganisms under stressful environments (62, 63). In addition, the presence of a motility system like type IV pilus-dependent twitching in all the MAGs and flagellar motor system in subgroups 3 and A may help cells migrate to more favorable niches. To summarize, these strategies in response to stress depicted in these MAGs are known to be widely used by other oligotrophic microbes (58, 64).

### Evolutionary history.

**FIG 4:**
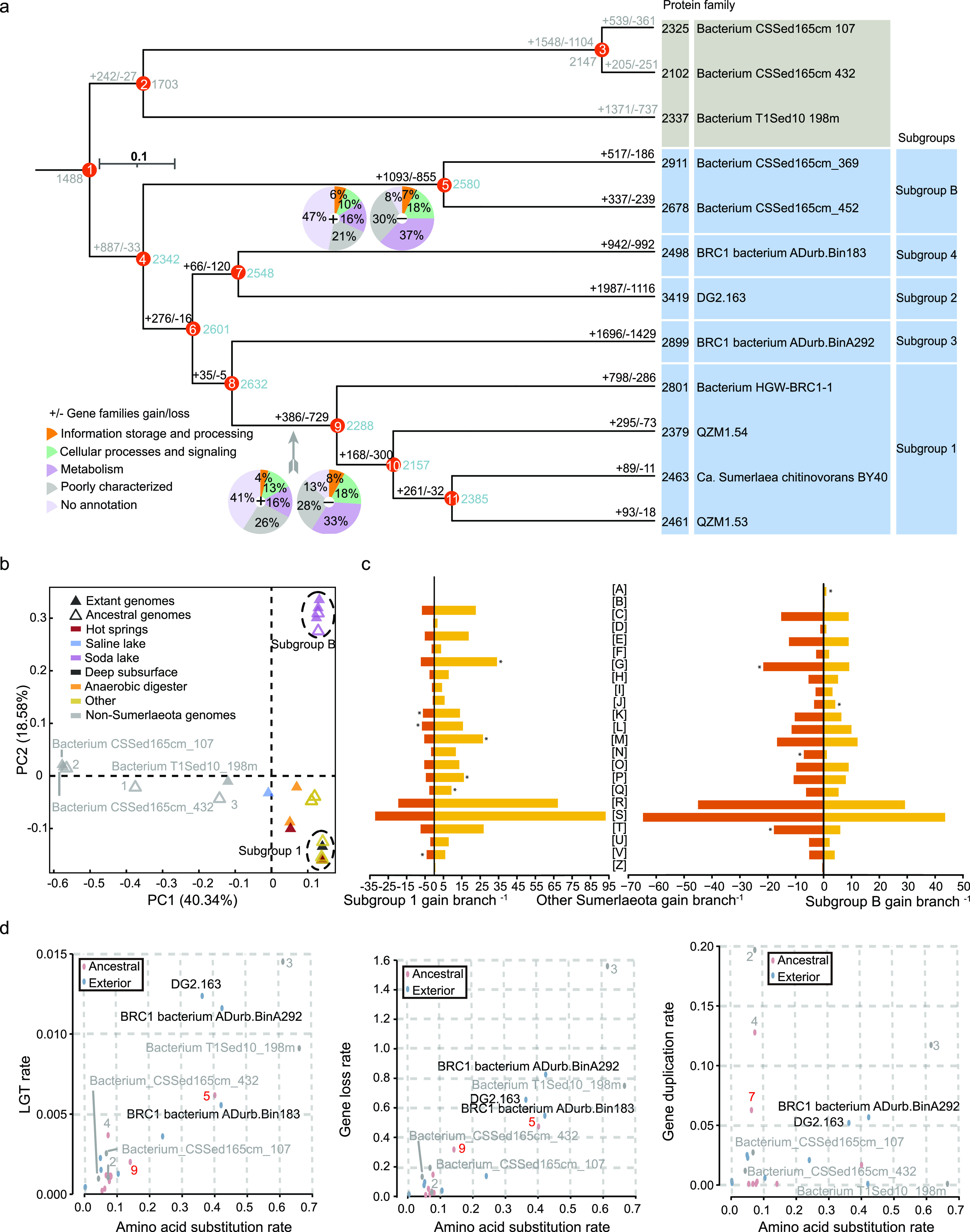
Ancestral genome content reconstruction and diverse rate analyses. (a) Ancestral state reconstruction of the Sumerlaeota. This Bayesian tree is generated based on a concatenation of 16 ribosomal proteins using MrBayes. The pie chart shows the fractions of gained or lost genes by COG categories. A list of gained and lost genes for each node is shown in Data Set S1h. (b) PCoA plot with Bray-Curtis dissimilarity based on COG categories of the ancestral and extant genomes of the Sumerlaeota. (c) Gene families gained per branch in subgroup B and other Sumerlaeota branches. Asterisks indicate significant differences in proportions using the Xipe analysis (*P < *0.01). The horizontal axis shows the number of gene families gained per branch for each COG category. [A], RNA processing and modification; [B], chromatin structure and dynamics; [C], energy production and conversion; [D], cell cycle control, cell division, chromosome partitioning; [E], amino acid transport and metabolism; [F], nucleotide transport and metabolism; [G], carbohydrate transport and metabolism; [H], coenzyme transport and metabolism; [I], lipid transport and metabolism; [J], translation, ribosomal structure and biogenesis; [K], transcription; [L], replication, recombination and repair; [M], cell wall/membrane/envelope biogenesis; [N], cell motility; [O], posttranslational modification, protein turnover; [P], inorganic ion transport and metabolism; [Q], secondary metabolites biosynthesis, transport, and catabolism; [R], general function prediction only; [S], function unknown; [T], signal transduction mechanisms; [U], intracellular trafficking, secretion, and vesicular transport; [V], defense mechanisms; [Z], cytoskeleton. (d) Analyses of lateral gene transfer (LGT) rate, gene loss rate, and gene duplication rate versus amino acid substitution rate on the Sumerlaeota branches.

The Sumerlaeota represent an evolutionarily diverse bacterial lineage distributed in diverse environments. To decipher the flux of gene families in the Sumerlaeota, the birth-and-death model in COUNT was implemented based on a robust Bayesian phylogenomic tree (Fig. 4 and Fig. S6). The common ancestor was inferred to contain 2,342 orthologous genes (Fig. 4a and Fig. S4 and Data Set S1g), including those encoding the complete pathway of chitin degradation, terminal oxidases (e.g., *aa*_3_-type cytochrome *c* oxidase and cytochrome *bd* ubiquinol oxidase), terminal oxidoreductases of the anaerobic respiratory (e.g., NrfAH-like nitrite reductase), and enzymes involved in fermentation (e.g., phosphate acetyltransferase, acetate kinase, and lactate dehydrogenase) (Data Set S1h). This finding suggested a chemoorganotrophic and facultatively anaerobic lifestyle for this common ancestor. Note that rare gene gain and loss events occurred at the branch leading to node 8 (Fig. 4a), suggesting the lack of niche expansion and population size effects during this period (65–67). Afterward, large gene gain and loss events occurred at the branches leading to nodes 5 and 9, which shaped genome contents of subgroups B and 1, respectively. Taking node 5 as an example, some important genes were gained, including those encoding sulfite reductase (NADPH), flavoprotein alpha-component, nitroreductase, and l-lactate dehydrogenase; meanwhile, the key gene *glk*, encoding glucokinase in the Embden-Meyerhof-Parnas (EMP) pathway, was lost (Data Set S1i). These findings likely result in metabolic differentiation between subgroup B and the other subgroups (Fig. 4b). Considering the completeness of all four genomes belonging to subgroup B, it was extremely unlikely that all the *glk* genes were absent, yet we could not completely rule out that the loss of these genes was due to the incomplete genomes. For node 9, some important genes, such as glycerophospholipid transport system (*mla*), were lost, potentially characterizing the metabolic feature of subgroup 1 (Fig. 2 and Data Set S1h). Notably, compared with large gene gain events, larger loss events occurred at the branch to the last common ancestor of subgroup 1 (node 9), likely due to niche expansion and population size effects (65–67). At the tips of the phylogeny, gene gain and loss events occurred at a larger scale along the branches, leading to extant organisms of subgroups 2, 3, and 4, which might reshape their genome contents, leading to the metabolic diversification of Sumerlaeota. In particular, several genes required for the assembly and motility of flagella (e.g., hook-associated protein 2 and flagellar protein FliS) were gained for subgroup 3, while genes related to dissimilatory sulfate reduction (e.g., adenylylsulfate reductase and dissimilatory sulfite reductase) were gained for subgroup 2. Such different gene gains might account for niche differentiation of these taxa. Thus, three significant evolutionary stages were predicted: the first was manifested as relatively rare gene flux along the branches leading to nodes 6, 7, and 8; the second was summarized as massive gene flux along the branches leading to nodes 5 and 9; and the last occurred more recently along the branches leading to extant Sumerlaeota organisms.

10.1128/mBio.00350-21.6 FIG S6 Bayesian tree of the Sumerlaeota reconstructed based on a concatenation of 16 riboproteins. Download FIG S6, EPS file, 1.3 MB. Copyright © 2021 Fang et al. 2021 Fang et al. https://creativecommons.org/licenses/by/4.0/ This content is distributed under the terms of the Creative Commons Attribution 4.0 International license.

Cluster analysis based on Clusters of Orthologous Groups of proteins (COGs) of ancestral and extant genomes affiliated with Sumerlaeota revealed significant metabolic divergence among subgroup B, subgroup 1, and the others (Fig. 4b), suggesting significant ecological transitions (67, 68). During evolutionary innovation in the Sumerlaeota lineages, laterally acquired gene families were biased toward “RNA processing and modification,” “replication, recombination, and repair,” and “posttranslational modification, protein turnover, chaperones” for subgroup B and “carbohydrate transport and metabolism,” “cell motility,” and “signal transduction mechanisms” for the others (Fig. 4c). Considering that members of subgroup B inhabit soda lakes, they may use RNA processing and modification to protect RNA from hydrolysis by alkali (69, 70). In addition, compared with the others, lateral acquisitions were biased toward “defense mechanisms,” “transcription,” and “replication, recombination, and repair” for subgroup 1 (Fig. 4c). Of these, 29 acquired genes belonging to defense mechanisms, and 18 and 8 were related to the restriction/modification system and multidrug efflux pumps, respectively, likely conferring on members of subgroup 1 the ability to resist antibiotics and viruses. Therefore, this nonrandom acquisition was indicative of niche differentiation and adaptive evolution for the Sumerlaeota populations.

This study further revealed a complex evolutionary path for the genome content of Sumerlaeota (Fig. 4a). Intriguingly, the rates of gene gain and loss followed molecular clocks for both ancestral and extant branches, because they showed significant correlations with the amino acid substitution rate (averaging 0.02 gene family gain and 1.58 deletions per amino acid substitution for MrBayes), respectively (both *P < *0.001; Fig. 4d and Data Sets S1i and j). These findings indicated that both gene gain and loss occurred at constant rates for both ancestral and extant branches. In contrast, the gene duplication rate did not follow a molecular clock for the Sumerlaeota branches (*P > *0.05; Data Set S1j).

### Conclusions.

This study deeply explores the biology of Sumerlaeota (the former BRC1). 16S rRNA gene-based analyses showed the global distribution of Sumerlaeota that are specially adapted to some harsh, nutrient-limited environments (e.g., cold arid desert soils and deep-sea basin surface sediments). Metabolic reconstruction indicated that the Sumerlaeota, which possibly originated from facultatively anaerobic ancestors, appeared capable of chemoorganotrophy and chemolithotrophy using a variety of carbon sources, nitrogen sources, phosphorus sources, and electron donors, suggesting that they play the role of scavengers for complex organics in nature. The finding is also evidenced by the confirmed chitinolytic activities (9), and other metagenomic-based physiological characteristics of this mysterious bacterial lineage remain to be verified. Such versatile metabolic potentials are considered adaptive strategies for Sumerlaeota to survive in diverse environments. Moreover, physiological deviation on different Sumerlaeota orders is likely attributed to their different evolutionary paths. Overall, in-depth analyses of these MAGs further advance our understanding of the environmental distribution, possible ecological roles, and evolutionary history of this elusive bacterial lineage, providing an important foundation for future Sumerlaeota study.

## MATERIALS AND METHODS

### Sampling, DNA extraction, and sequencing.

The sampling expedition took place in August 2019. Surface sediment samples were collected from the Quzhuomu hot spring (QZM1; 28°24.4′ N, 91°81.1′ E), downstream of Daggyai hot spring (DG2; 29°59.9′ N, 85°75.1′ E), and Xiaochaidan Lake (XCDL20; 37°27.3′ N, 95°28.5′ E), China (see Fig. S1 in the supplemental material). These sediments were collected into 50-ml sterile centrifuge tubes and were stored immediately on dry ice until arrival in the laboratory. Physicochemical parameters were measured either *in situ* or in the laboratory, as previously described (71) and listed in Data Set S1a. In brief, the pH, temperature (T), and concentration of dissolved oxygen (DO), Fe^2+^, and S^2−^ were measured *in situ* with a temperature/pH probe (DR850; HACH Company, CO) and Hach kits, respectively. Total organic carbon (TOC) and dissolved organic carbon (DOC) were measured with a Multi N/C 2100S analyzer (Analytik, Jena, Germany). The concentrations of major ions (e.g., K^+^, Ca^2+^, Na^+^, Mg^2+^, Cl^−^, and SO_4_^2−^) were determined by using a Dionex DX 600 ion chromatograph (Dionex, USA). Genomic DNA was extracted from 10 g of each sediment sample using our modified phenol-chloroform method (72). Standard shotgun libraries of 300 bp in insert size were conducted at the Guangdong Magigene Company and then were sequenced on an Illumina HiSeq 4000 platform (paired-end 150-bp mode).

### Metagenomic analysis.

Raw reads were pretreated using a custom Perl script and Sickle as we previously reported (22). The resultant high-quality reads for each sample then were assembled independently using SPAdes (version 3.11.0) with the parameters listed in Data Set S1b. The scaffolds were binned based on the tetranucleotide frequencies and scaffold coverage using MetaBAT (version 2.12.1) (73) with the parameters “-m 2000 –unbinned.” The preliminary classification of all bins was confirmed using the Genome Taxonomy Database Toolkit (GTDB-Tk) (19), and four genome bins belonging to the Sumerlaeota were selected. As described previously (22), they were reassembled using the recruited reads through BBMap (74) and were examined manually to remove possible contamination. Their completeness, contamination, and strain heterogeneity were evaluated by using CheckM (75). These curated genomes were used for the subsequent analyses, including functional annotation, phylogenomic and phylogenetic analyses, metabolic inference, and ancestral state reconstruction.

### Genome annotation and metabolic reconstruction.

Gene prediction was performed using Prodigal with the “-p single” option for each genome (76), and then protein-coding genes were annotated based on comparisons with the NCBI-nr, KEGG, EggNOG, and Pfam databases using DIAMOND with an E value of ≤1e−5 (77). Carbohydrate-active enzymes were identified through the dbCAN2 meta server. Metabolic pathways for each bin were reconstructed based on the manually curated gene annotation.

### Phylogenomic and phylogenetic analyses.

Sixteen ribosomal proteins (riboproteins) (78) and 35 marker proteins (79) identified from Sumerlaeota genomes and representative genomes collected from the GTDB database were individually aligned using MAFFT with the parameters “–localpair –maxiterate 1000” (80) and then were filtered with TrimAL with the parameters “-gt 0.95 -cons 50” (81). The 16 riboprotein-based and 35 marker protein-based phylogenomic trees were constructed using IQ-TREE with the parameters “LG+F+I+G4 -alrt 1000 -bb 1000” and “LG+I+G4 -alrt 1000 -bb 1000,” respectively (82). Moreover, 16S rRNA gene sequences from Sumerlaeota genomes and environmental 16S rRNA gene sequences were aligned using SINA (83), and then the alignment was filtered by TrimAL. The 16S rRNA gene-based phylogenetic tree was constructed using IQ-TREE with the parameters “LG+I+G4 -alrt 1000 -bb 1000.” Furthermore, a phylogenetic tree of chitinases was constructed using IQ-TREE with the parameters “WAG+F+G4.” Homology modeling of the protein was done using the Phyre2 web tool (http://www.sbg.bio.ic.ac.uk/phyre2) via the Hidden Markov Method (84). The three-dimensional (3D) structure models for type A and B chitinases were developed based on similarities to templates 4txgA and 6bt9B under “intensive” mode. The final predicted model was submitted to the 3DLigandSite server (http://www.sbg.bio.ic.ac.uk/3dligandsite/) to predict the potential binding site (85). In addition, the 426 DsrAB protein sequences were collected from the GTDB database and a previous study (51), which were aligned and filtered as mentioned above. The DsrAB protein-based phylogenetic tree was also built using IQ-TREE with the parameters “LG+I+G4 -alrt 1000 -bb 1000.” The generated newick files for trees were uploaded to iTOL for visualization and formatting (86).

### Comparative genomics.

The OrthoANIu and AAI values among these 16 genomes were calculated (87, 88). For COUNT analysis, only 9 genomes with estimated completeness greater than 85% were kept, and clusters of homologous proteins were constructed. An all-against-all genome BLAST was carried out to yield reciprocal best BLAST hits (rBBHs) with threshold E values of <1e−10 and local amino acid identity of ≥25%. The Needleman-Wunsch algorithm in EMBOSS v6.5.7 was employed to align these protein pairs with a threshold global amino acid identity of ≥25%, and MCL (-I 1.4) was used to generate protein clusters based on rBBHs (89). A total of 16,085 protein families were obtained, of which 10,179 were singletons. Bayesian inference analysis was implemented by MrBayes v3.2.6 (90) with the following parameters: 8 independent chains, 2 simultaneous runs, 2 million generations, 0.25 burn-in fraction, 8 rate categories for the gamma distribution, a heating factor of 0.15, and LG model with empirical amino acid frequencies and invgamma rates determined by ProtTest 3 (91). It was considered a good indication of convergence that the average standard deviations of split frequencies were less than 0.01 using Markov chain Monte Carlo analysis. The evolutionary history of Sumerlaeota was inferred using COUNT v9.1106 with maximum likelihood (ML) birth-and-death models (92). The likelihood of the phyletic pattern (vector of observed family sizes at terminal taxa) was maximized under a gain-loss-duplication model with a Poisson distribution at the root and 4:1:1:4 gamma categories for the edge length and loss, gain, and duplication rates, respectively. Family sizes and lineage-specific events (including gains, losses, expansions, and contractions) were computed based on posterior probabilities in the optimized ML model. The convergence criteria for the optimization were set to 1,000 rounds with a likelihood threshold of 0.01. These inferred rates of gene transfer, loss, and duplication were plotted against amino acid substitution rate on the branches. Additionally, a significant (*P < *0.01) enrichment of specific COG categories in the uniquely shared genes among the corresponding MAGs was determined based on 20,000 repetitions and a sample size of 10,000 by the Xipe analysis.

### Data availability.

The four genomes retrieved in this study have been deposited in the NCBI database with accession numbers JADFCT000000000-JADFCW000000000.
