# Supplementary material for: Casting Light on the Adaptation Mechanisms and Evolutionary History of the Widespread Sumerlaeota
Source: mBio. 2021 Mar 30;12(2):e00350-21. doi: 10.1128/mBio.00350-21 (PMC8092238; doi:10.1128/mBio.00350-21)
Supplement: TEXT S1 [file mBio.00350-21-s0001.docx]

Supplementary Information

**Casting light on the adaptation mechanisms and evolutionary history of the widespread Sumerlaeota**

Yun Fang^a,b^, Yang Yuan^c^, Jun Liu^c^, Geng Wu^a,*^, Jian Yang^a^, Zhengshuang Hua^d^, Jibin Han^e^, Xiying Zhang^e^, Wenjun Li^b,c^, Hongchen Jiang^a,b,*^

^a^State Key Laboratory of Biogeology and Environmental Geology, China University of Geosciences, Wuhan, China;

^b^State Key Laboratory of Desert and Oasis Ecology, Xinjiang Institute of Ecology and Geography, Chinese Academy of Sciences, Urumqi, China.

^c^State Key Laboratory of Biocontrol, Guangdong Provincial Key Laboratory of Plant Resources and Southern Marine Science and Engineering Guangdong Laboratory (Zhuhai), School of Life Sciences, Sun Yat-Sen University, Guangzhou, China;

^d^Department of Biological Sciences, Dartmouth College, Hanover, USA;

^e^Key Laboratory of Salt Lake Geology and Environment of Qinghai Province, Qinghai Institute of Salt Lakes, Northwest Institute of Eco-Environment and Resources, Chinese Academy of Sciences, Xining, China;

*Correspondence:

Hongchen Jiang, jiangh@cug.edu.cn;

Geng Wu, wugeng@cug.edu.cn.

**References in Data Set S1d**

1. Harris JK, Caporaso JG, Walker JJ, Spear JR, Gold NJ, Robertson CE. Phylogenetic stratigraphy in the Guerrero Negro hypersaline microbial mat. ISME J 2013; 7: 50-60.
2. Chiellini C, Munz G, Petroni G, Lubello C, Mori G, Verni F, et al. Characterization and comparison of bacterial communities selected in conventional activated sludge and membrane bioreactor pilot plants: a focus on *Nitrospira* and *Planctomycetes* bacterial phyla. Curr Microbiol 2013; 67: 77-90.
3. Park H, Rosenthal A, Ramalingam K, Fillos J, Chandran K. Linking community profiles, gene expression and N-removal in anammox bioreactors treating municipal anaerobic digestion reject water. Environ Sci Technol 2010; 44: 6110-6116.
4. Riviere D, Desvignes V, Pelletier E, Chaussonnerie S, Guermazi S, Weissenbach J et al. Towards the definition of a core of microorganisms involved in anaerobic digestion of sludge. ISME J 2009; 3: 700-714.
5. Schauer R, Bienhold C, Ramette A, Harder J. Bacterial diversity and biogeography in deep-sea surface sediments of the South Atlantic Ocean. The ISME J 2010; 4: 159-170.
6. Lesaulnier C, Papamichail D, McCorkle S, Ollivier B, Skiena S, Taghavi S et al. Elevated atmospheric CO_2_ affects soil microbial diversity associated with trembling aspen. Environ Microbiol 2008; 10: 926-941.
7. Derakshani M, Lukow T, Liesack W. Novel bacterial lineages at the (sub) division level as detected by signature nucleotide-targeted recovery of 16S rRNA genes from bulk soil and rice roots of flooded rice microcosms. Appl Environ Microbiol 2001; 67: 623-631.
8. Li T, Wang P. Biogeographical distribution and diversity of bacterial communities in surface sediments of the South China Sea. J Microbiol Biotechnol 2013; 23: 602-613.
9. Elshahed MS, Youssef NH, Spain AM, Sheik C, Najar FZ, Sukharnikov LO et al. Novelty and uniqueness patterns of rare members of the soil biosphere. Appl Environ Microbiol 2008; 74: 5422-5428.
10. Isenbarger TA, Finney M, Ríos-Velázquez C, Handelsman J, Ruvkun G. Miniprimer PCR, a new lens for viewing the microbial world. Appl Environ Microbiol 2008; 74: 840-849.
11. Santelli CM, Orcutt BN, Banning E, Bach W, Moyer CL, Sogin ML et al. Abundance and diversity of microbial life in ocean crust. Nature 2008; 453: 653-656.
12. Field EK, D'Imperio S, Miller AR, VanEngelen MR, Gerlach R, Lee BD et al. Application of molecular techniques to elucidate the influence of cellulosic waste on the bacterial community structure at a simulated low-level-radioactive-waste site. Appl Environ Microbiol 2010; 76: 3106-3115.
13. Bouali M, Pelletier E, Chaussonnerie S, Le Paslier D, Bakhrouf A, Sghir A. Characterization of rhizosphere prokaryotic diversity in a horizontal subsurface flow constructed wetland using a PCR cloning-sequencing based approach. Appl Microbiol Biotechnol 2013; 97: 4221-4231.
14. Bouali M, Zrafi I, Bakhrouf A, Chaussonnerie S, Sghir A. Bacterial structure and spatiotemporal distribution in a horizontal subsurface flow constructed wetland. Appl Microbiol Biotechnol 2014; 98: 3191-3203.
15. Watanabe T, Kojima H, Fukui M. Identity of major sulfur-cycle prokaryotes in freshwater lake ecosystems revealed by a comprehensive phylogenetic study of the dissimilatory adenylylsulfate reductase. Sci Rep 2016; 6: 36262.
16. Czarnetzki AB, Tebbe CC. Diversity of bacteria associated with *Collembola*–a cultivation-independent survey based on PCR-amplified 16S rRNA genes. FEMS Microbiol Ecol 2004; 49: 217-227.
17. Kim JS, Crowley DE. Microbial diversity in natural asphalts of the Rancho La Brea Tar Pits. Appl Environ Microbiol 2007; 73: 4579-4591.
18. Lau MCY, Aitchison JC, Pointing SB. Bacterial community composition in thermophilic microbial mats from five hot springs in central Tibet. Extremophiles 2009; 13: 139-149.
19. Williamson KE, Kan J, Polson SW, Williamson SJ. Optimizing the indirect extraction of prokaryotic DNA from soils. Soil Biol Biochem 2011; 43: 736-748.
20. Direito SOL, Ehrenfreund P, Marees A, Staats M, Foing B, Röling WF. A wide variety of putative extremophiles and large beta-diversity at the Mars Desert Research Station (Utah). Int J Astrobiol 2011; 10: 191.
21. Tian W, Sun Q, Xu D, Zhang Z, Chen D, Li C et al. Succession of bacterial communities during composting process as detected by 16S rRNA clone libraries analysis. Int Biodeterior Biodegradation 2013; 78: 58-66.
22. Chen Y, Wen X, Sun Y, Zhang J, Wu W, Liao Y et al. Mulching practices altered soil bacterial community structure and improved orchard productivity and apple quality after five growing seasons. Sci Hortic 2014; 172: 248-257.
23. Freitag TE, Prosser JI. Community structure of ammonia-oxidizing bacteria within anoxic marine sediments. Appl Environ Microbiol 2003; 69: 1359-1371.
24. Chouari R, Le Paslier D, Dauga C, Daegelen P, Weissenbach J, Sghir A. Novel major bacterial candidate division within a municipal anaerobic sludge digester. Appl Environ Microbiol 2005; 71: 2145-2153.
25. Kong H H, Oh J, Deming C, Conlan S, Grice EA, Beatson MA et al. Temporal shifts in the skin microbiome associated with disease flares and treatment in children with atopic dermatitis. Genome Res 2012; 22: 850-859.
26. Karst SM, Dueholm MS, McIlroy SJ, Kirkegaard RH, Nielsen PH, Albertsen M. Retrieval of a million high-quality, full-length microbial 16S and 18S rRNA gene sequences without primer bias. Nat Biotechnol 2018; 36: 190-195.
