# Supplementary figures and images for: Casting Light on the Adaptation Mechanisms and Evolutionary History of the Widespread Sumerlaeota
Source: mBio. 2021 Mar 30;12(2):e00350-21. doi: 10.1128/mBio.00350-21 (PMC8092238; doi:10.1128/mBio.00350-21)

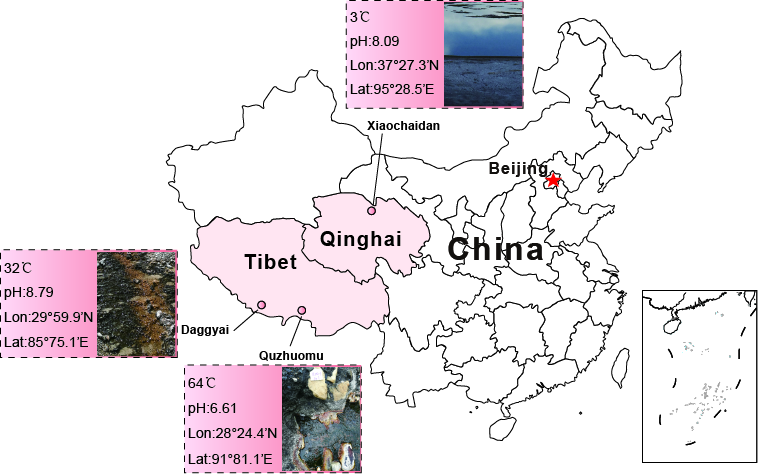

Supplement: FIG S1 [file mBio.00350-21-sf001.tif]

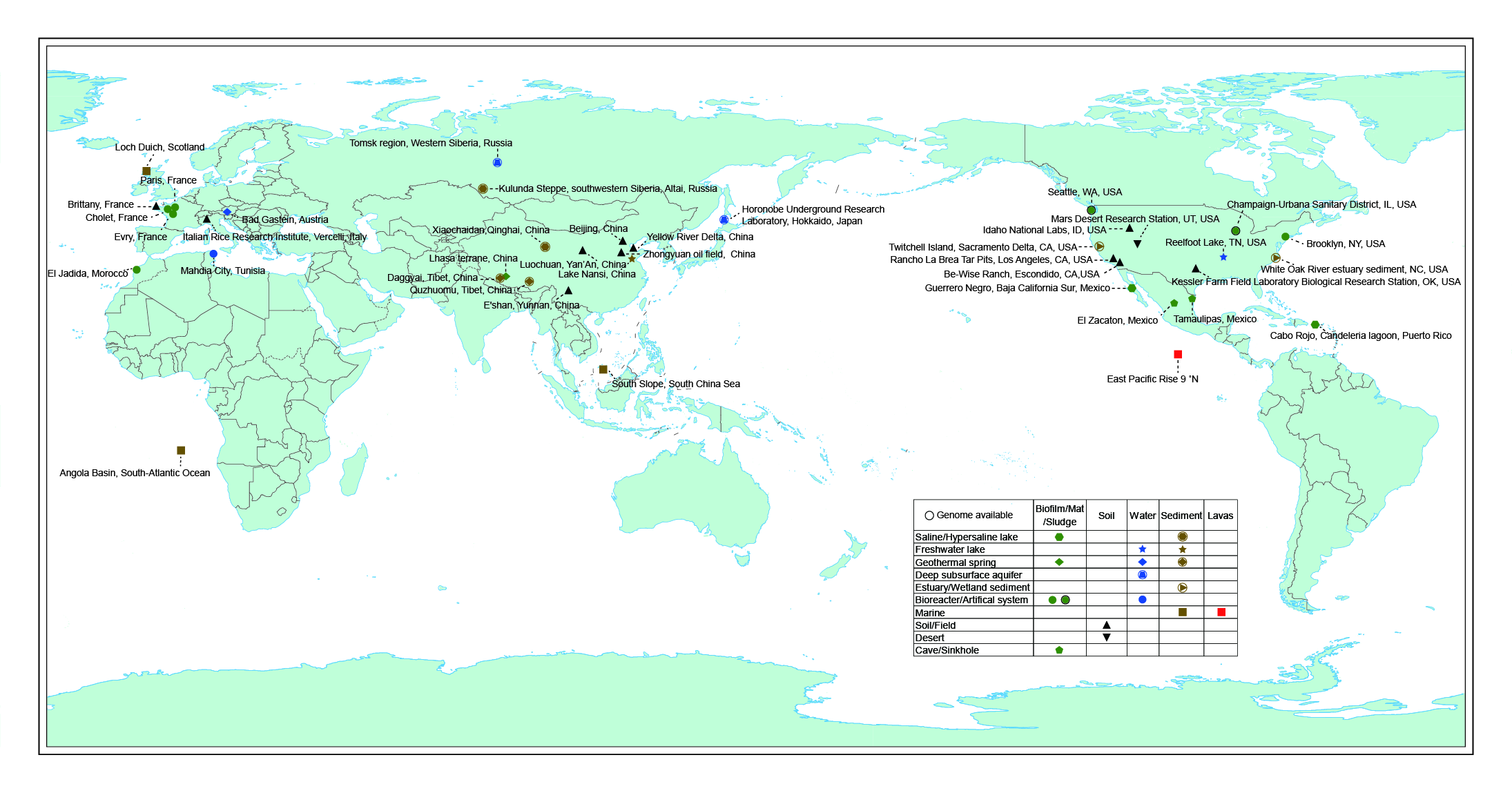

Supplement: FIG S2 [file mBio.00350-21-sf002.tif]
